# PD-1/CTLA-4 antibody AK104 in advanced solid tumors after PD/PD-L1 treatment failure: A retrospective cohort study

**DOI:** 10.1097/MD.0000000000041548

**Published:** 2025-03-07

**Authors:** Jun Yi, Huangpeng Yu, Yun Shu, Yingen Deng

**Affiliations:** a Department of Oncology, The Third People’s Hospital of Jiujiang, Jiujiang, Jiangxi Province, China; b Department of Pharmacy, The Third People’s Hospital of Jiujiang, Jiujiang, Jiangxi Province, China.

**Keywords:** advanced solid tumors, AK104, efficacy, PD-1/CTLA-4 antibody, safety, tolerability

## Abstract

AK104 is a novel antibody targeting programmed cell death protein 1 (PD-1)/cytotoxic T-lymphocyte-associated protein 4. This study aimed to evaluate the safety, tolerability, and efficacy of AK104 in treating patients with advanced solid tumors who failed prior programmed cell death protein 1/programmed death-ligand 1 (PD/PD-L1) therapies. Clinical data from 135 patients with advanced solid tumors who failed PD/PD-L1 therapies were retrospectively analyzed. Patients received AK104 at a dose of 6 mg/kg every 2 weeks. Baseline demographic characteristics, clinical outcomes, adverse reactions, overall survival, progression-free survival, and quality of life assessments were analyzed. Following AK104 treatment, 17.78% of patients achieved a partial response, while 80.74% experienced stable disease, resulting in a disease control rate of 98.52%. The 1- and 2-year overall survival rates were 48.15% and 31.11%, while progression-free survival rates at 6 months and 1 year were 53.33% and 28.15%, respectively. Post-treatment, significant improvements in the quality-of-life scores, as assessed by the European Organisation for Research and Treatment of Cancer Quality of Life Questionnaire-Core 30 and EuroQol 5 Dimensions Visual Analog Scale, were observed post-treatment. Immune-related adverse events were common, affecting 85.19% of patients, with diarrhea, enteritis, pneumonia, and thyroid dysfunction being the most frequently reported. AK104 demonstrated the ability to induce clinical responses, extend survival, and enhance quality of life in patients with advanced solid tumors who had previously failed PD-1/PD-L1 therapies, underscoring its potential as a promising therapeutic option. However, the high incidence of immune-related adverse events necessitates vigilant monitoring and management to maximize its clinical utility. Further prospective studies are warranted to validate and extend these findings in broader patient populations.

## 1. Introduction

Advanced solid tumors present a significant challenge in modern oncology, contributing substantially to global morbidity and mortality.^[1–3]^ Despite the availability of diverse therapeutic modalities, including surgery, chemotherapy, and radiation therapy, the prognosis for patients with advanced solid tumors remains poor.^[4–6]^ This underscores the urgent need to explore novel treatment strategies to improve clinical outcomes.^[7–9]^ The advent of immune checkpoint inhibitors targeting programmed cell death protein 1 (PD-1) and programmed death-ligand 1 (PD-L1) has marked a breakthrough in cancer therapy, harnessing the immune system to combat malignant tumors.^[10,11]^ However, a significant proportion of patients fail to derive sustained benefit from PD/PD-L1 therapies, emphasizing the need for alternative treatment options for this challenging patient population.

The development of PD-1/PD-L1 inhibitors has significantly transformed the management of various malignancies, enabled durable responses and improved overall survival in some patients.^[12–14]^ These inhibitors enhance the host immune system’s antitumor activity and counteract tumor-mediated immune evasion.^[15–17]^ However, the efficacy of PD-1/PD-L1 blockade is limited by both primary and acquired resistance, leading to disease progression in a significant proportion of patients.^[18–20]^ Moreover, PD/PD-L1 therapies are associated with immune-related adverse events, which compromise long-term tolerability and can lead to treatment discontinuation in some cases.^[21–23]^ These challenges highlight the urgent need for alternative immunotherapeutic strategies capable of overcoming resistance mechanisms and improving outcomes in patients with advanced solid tumors.

AK104, a novel immune checkpoint inhibitor targeting both PD-1 and cytotoxic T-lymphocyte-associated protein 4 (CTLA-4), represents a promising advancement in cancer immunotherapy.^[24,25]^ By simultaneously targeting these critical immune checkpoints, AK104 offers a unique mechanism of action with the potential to enhance antitumor immune responses and overcome resistance to PD/PD-L1 therapies.^[26]^ Preclinical and early clinical data suggest that AK104 may exert synergistic effects on T cell activation and effector function, leading to enhanced antitumor activity in preclinical models and early-phase clinical trials.^[27]^ Furthermore, the safety and tolerability profile of AK104 appears favorable, with manageable immune-related adverse events, offering a potential advantage over existing PD-1/PD-L1 inhibitors.^[25]^

The failure of PD-1/PD-L1 inhibitors in a substantial proportion of patients highlights the need for alternative immunotherapeutic strategies in advanced solid tumors. AK104 holds promise as a viable treatment option for patients who have experienced disease progression or intolerable toxicities following PD/PD-L1 therapies. The dual blockade of PD-1 and CTLA-4 by AK104 provides a rational approach to reinvigorate antitumor immunity in patients who have failed prior PD/PD-L1 treatments, potentially overcoming resistance and fostering durable clinical responses.^[12,18]^ Therefore, a comprehensive evaluation of the safety, tolerability, and efficacy of AK104 in this patient population is crucial to elucidate its potential role in managing advanced solid tumors.

Therefore, investigating AK104 in the context of advanced solid tumors that have failed prior PD/PD-L1 therapies is warranted, given the unmet medical needs of this patient population and the potential benefits of dual PD-1/CTLA-4 blockade. This study aimed to evaluate the safety, tolerability, and efficacy of AK104 in this context, so as to provide insights into the therapeutic landscape of advanced solid tumors and informing future clinical practice.

## 2. Materials and methods

### 2.1. Research object

This retrospective cohort study aimed to assess the safety, tolerability, and efficacy of AK104, a PD-1/CTLA-4 antibody, in the management of patients with advanced solid tumors who had previously experienced treatment failure with PD/PD-L1 therapies, defined as disease progression or the presence of significant adverse events leading to treatment discontinuation, as documented in the medical records. A total of 135 patients, comprising 75 males and 60 females, aged between 43 and 66 years with a mean age of 56.51 ± 6.72 years, admitted to our institution from January 2021 to December 2022, were included in the analysis.

The retrospective analyses of patient information were approved by the Ethic Committee of the Third People’s Hospital of Jiujiang, approval number No. JJSDSYY-LLWYH-2023050, under the guideline of STROBE. Informed consent was waived for this retrospective study due to the exclusive use of de-identified patient data, which posed no potential harm or impact on patient care.

### 2.2. AK104 treatment regimen

In this study, the PD-1/CTLA-4 bispecific antibody used is Candonilimab (code name: AK104), marketed as Atyrso and manufactured by Kangfang Biotech (Specifications: 125 mg [10 mL]/vial). Candonilimab was administered at a dose of 6 mg/kg, extracted for injection and diluted in 100 mL of normal saline for intravenous infusion, resulting in a concentration range of 0.2 to 5.0 mg/mL. The diluted solution was gently inverted to ensure uniform mixing. The medication was then administered via intravenous infusion over approximately 60 ± 10 minutes. The dosage regimen involved administering 6 mg/kg every 2 weeks until disease progression or the occurrence of intolerable toxicity.

### 2.3. Inclusion and exclusion criteria

Inclusion criteria: patients with histologically confirmed advanced solid tumors^[6]^; patients who have previously failed PD/PD-L1 therapies; age between 18 and 75 years.

Exclusion criteria: patients with active, known, or suspected autoimmune disease; patients who have received systemic immunosuppressive therapy within a defined period before the study; history of allogeneic organ transplantation; known history of human immunodeficiency virus, hepatitis B, or hepatitis C infection; uncontrolled intercurrent illness including, but not limited to, ongoing or active infection, symptomatic congestive heart failure, unstable angina pectoris, cardiac arrhythmia, or psychiatric illness/social situations that would limit compliance with study requirements; known history of central nervous system metastases; Pregnancy or lactation.

### 2.4. Data collection

Retrospective data collection was conducted to gather baseline demographic characteristics, including age, gender, average disease duration, body mass index (BMI), Eastern Cooperative Oncology Group (ECOG) score, smoking and alcohol history, drug allergy history, and tumor types. Additionally, clinical efficacy outcomes, adverse reactions, overall survival, progression-free survival, quality of life assessments, and adverse events were systematically retrieved from patient medical records, treatment logs, and follow-up visits.

### 2.5. Outcome measures

The primary outcome measures included the safety and tolerability of AK104, as indicated by the incidence and severity of adverse events, especially immune-related adverse events. Additionally, the study assessed the efficacy of AK104 through response rates (complete response, partial response, stable disease, and progressive disease), overall survival, progression-free survival, and quality of life assessments.

### 2.6. Statistical analysis

The data were analyzed using SPSS 25.0 statistical software. Descriptive statistics for categorical data were expressed as [n (%)]. For sample sizes ≥ 40 and theoretical frequency (*T*) ≥ 5, the chi-square test was employed using the basic formula, with the test statistic denoted as χ^2^. In cases where the sample size was ≥ 40 but the theoretical frequency fell within the range 1 ≤ *T* < 5, the chi-square test correction formula was applied. For sample sizes < 40 or theoretical frequency *T* < 1, statistical analysis was conducted using the Fisher exact probability method. Continuous data conforming to a normal distribution were expressed as (mean ± standard deviation), while non-normally distributed data underwent variable transformation to achieve normal distribution for statistical analysis, utilizing the *t* test.

## 3. Results

### 3.1. Baseline demographic characteristics

A total of 135 patients with advanced solid tumors, who had previously failed PD-1/PD-L1 therapies, were enrolled in the study. Upon initiation of AK104 treatment, the patients had an average age of 56.51 years (±6.72) and a balanced gender distribution of 75 males and 60 females. The average disease duration prior to AK104 treatment was 2.12 months (±0.54), and the mean BMI was 26.21 (±4.12). Baseline ECOG scores averaged 1.56 (±0.51), reflecting relatively good functional status. The patients also had an average smoking history of 15.36 years (±8.26) and an alcohol consumption history of 10.34 years (±6.01). Additionally, 30 patients (22.22%) reported a history of drug allergies.

In terms of tumor types, all enrolled patients had advanced solid tumors, with the following distribution: non-small cell lung cancer, liver cancer, esophageal cancer, and stomach cancer each accounted for 22.22% of cases, while other tumor types represented 11.11%. These baseline characteristics provide a detailed overview of the study population and form a solid foundation for assessing the safety, tolerability, and efficacy of AK104 in patients with advanced solid tumors following failure of PD-1/PD-L1 therapies (Table 1).

**Table 1:** Baseline demographic characteristics of the patients.

Characteristic	After treatment
Age (yr)	56.51 ± 6.72
Gender (male/female)	75/60
Average disease duration (mo)	2.12 ± 0.54
BMI	26.21 ± 4.12
ECOG Score	1.56 ± 0.51
Smoking history (yr)	15.36 ± 8.26
Alcohol history (yr)	10.34 ± 6.01
Drug allergy history	
Yes	30 (22.22%)
No	105 (77.78%)
Tumor types (n, %)	
Non-small cell lung cancer	30 (22.22%)
Liver cancer	30 (22.22%)
Esophageal cancer	30 (22.22%)
Stomach cancer	30 (22.22%)
Others	15 (11.11%)

BMI = body mass index, ECOG = Eastern Cooperative Oncology Group.

### 3.2. Efficacy

**Figure 1.**
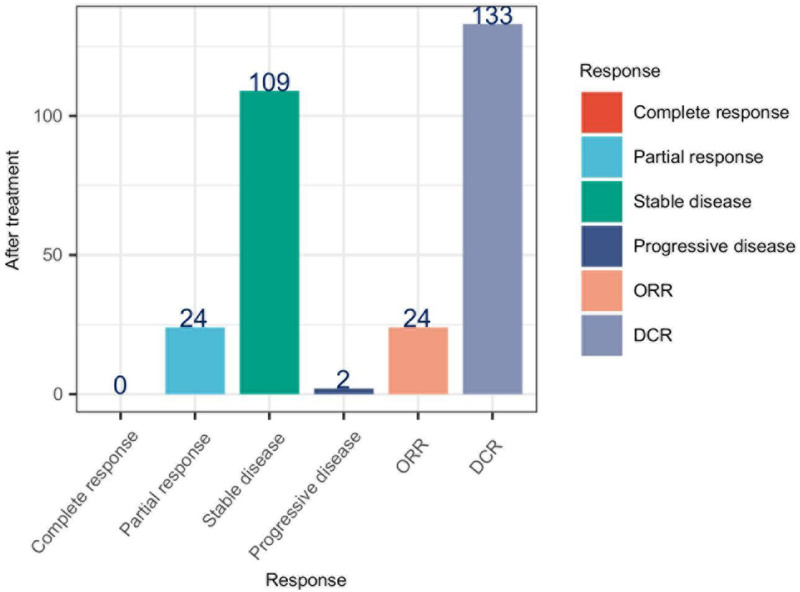
The efficacy of AK104 treatment in patients with advanced solid tumors who have previously failed PD/PD-L1 therapies. DCR = disease control rate, ORR = overall response rate, PD = programmed cell death protein, PD-L1 = programmed death-ligand 1.

The efficacy of AK104 in patients with advanced solid tumors previously unresponsive to PD-1/PD-L1 therapies was evaluated (Fig. 1). Following treatment, 24 out of 135 patients (17.78%) achieved a partial response, while 109 patients (80.74%) experienced stable disease, and 2 patients (1.48%) showed progressive disease. The overall response rate (ORR) was 17.78%, and the disease control rate (DCR) was 98.52%, reflecting a high proportion of patients who achieved either a partial response or disease stabilization with AK104 treatment. These findings highlight the potential efficacy of AK104 as a therapeutic option for advanced solid tumors, particularly in this heavily pretreated population.

### 3.3. Adverse reactions

**Figure 2.**
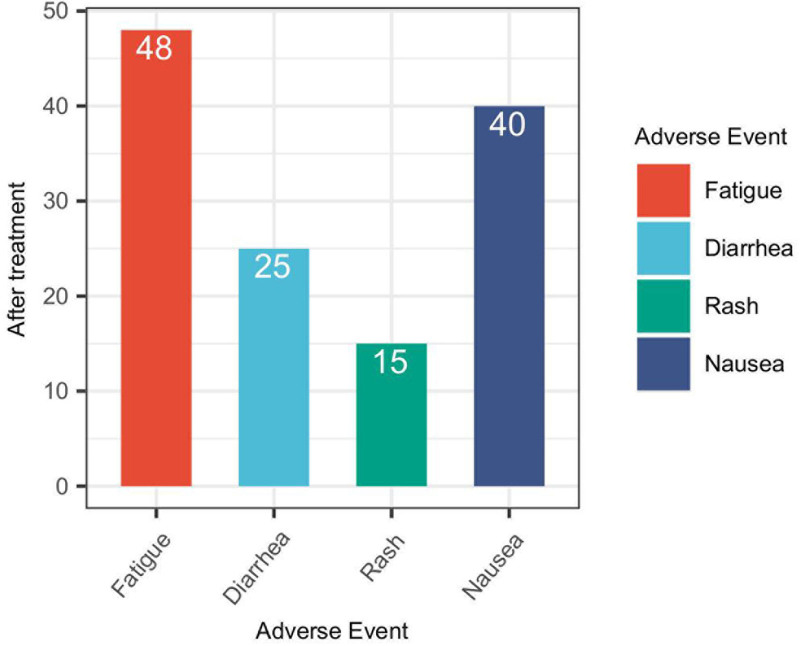
Adverse reaction after treatment with AK104.

Following treatment with AK104, several adverse events were observed among the patients. The most commonly reported adverse reactions were fatigue, experienced by 48 patients (35.56%), and nausea, reported by 40 patients (29.63%; Fig. 2). Diarrhea was documented in 25 patients (18.52%), and rash occurred in 15 patients (11.11%). These results suggest that fatigue and nausea are the most prevalent adverse events associated with AK104 treatment, underscoring the need for proactive monitoring and management of these symptoms in patients undergoing this therapeutic intervention.

### 3.4. Overall survival

**Figure 3.**
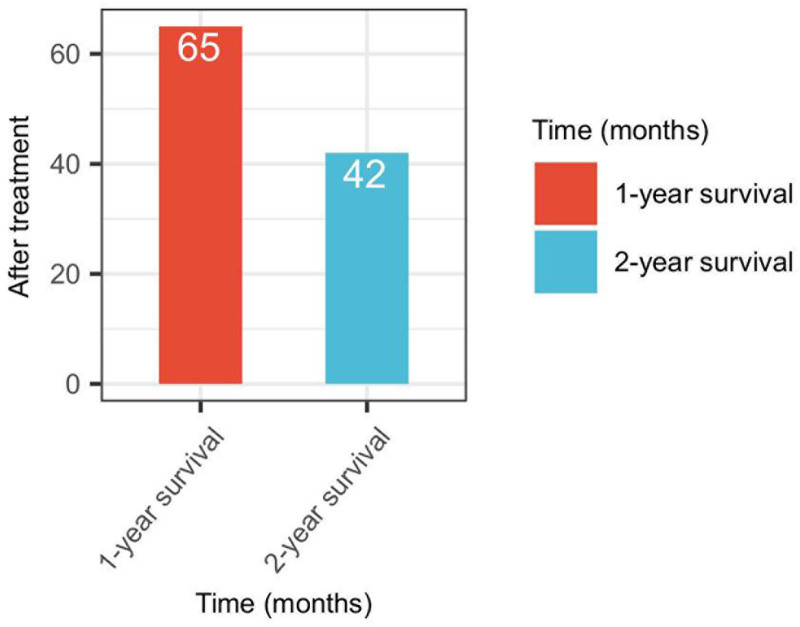
Overall survival after treatment with AK104.

The analysis of overall survival following treatment with AK104 revealed promising outcomes (Fig. 3). The 1-year survival rate was 48.15%, with 65 patients surviving beyond the first-year post-treatment. Notably, the 2-year survival rate was 31.11%, with 42 patients achieving long-term survival. These results suggest that AK104 provides sustained survival benefits for patients with advanced solid tumors who had previously failed PD-1/PD-L1 therapies. This highlights the potential of AK104 as an effective therapeutic option in this challenging clinical setting, warranting further exploration in larger cohorts and clinical trials.

### 3.5. Progression-free survival (PFS)

**Figure 4.**
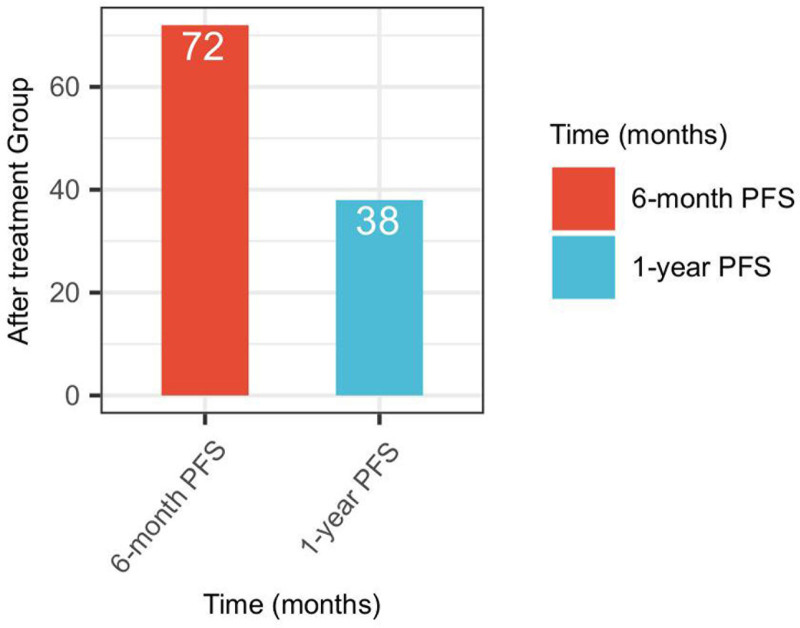
Progression free survival after treatment with AK104.

The analysis of progression-free survival (PFS) following treatment with AK104 yielded promising results (Fig. 4). The 6-month PFS rate was 53.33%, with 72 patients demonstrating disease control at this time point. Additionally, the 1-year PFS rate was 28.15%, with 38 patients maintaining progression-free status. These findings indicate that AK104 may provide substantial benefits in delaying disease progression, as reflected by the notable proportion of patients achieving sustained PFS at both 6 months and 1-year post-treatment.

### 3.6. Quality of life (QoL)

**Figure 5.**
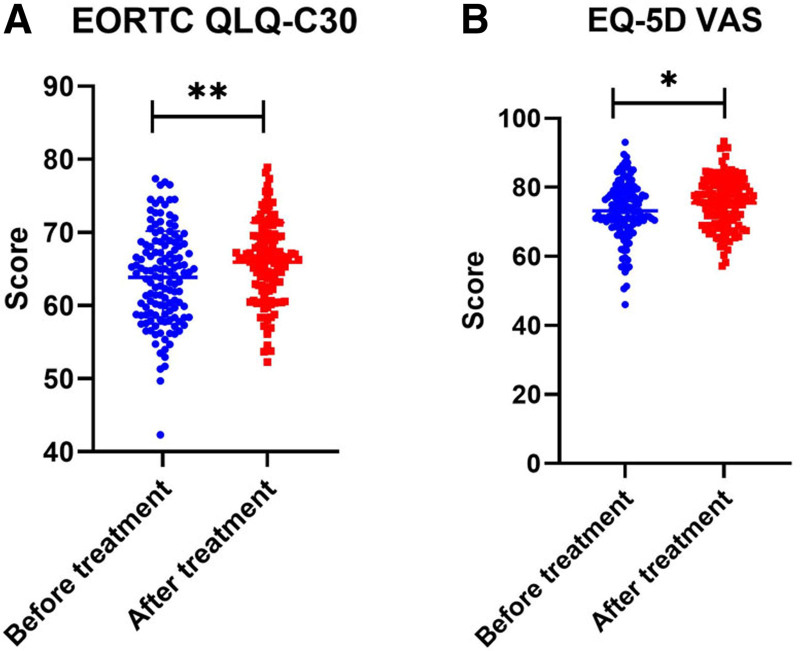
Quality of life after treatment with AK104. (A) EORTC QLQ-C30 score. (B) EQ-5D VAS score. * indicates *P* < .05, ** indicates *P* < .01, *** indicates *P* < .001. EORTC QLQ-C30 = European Organisation for Research and Treatment of Cancer Quality of Life Questionnaire-Core 30, EQ-5D VAS = Euroqol 5 Dimensions Visual Analogue Scale.

The assessment of AK104’s impact on the quality of life (QoL) in patients with advanced solid tumors revealed notable findings (Fig. 5). Following treatment, QoL showed a statistically significant improvement, as reflected by an increase in the European Organisation for Research and Treatment of Cancer Quality of Life Questionnaire-Core 30 score from 63.84 ± 6.36 before treatment to 65.92 ± 5.41 after treatment (*t* = 2.897, *P* = .004). Similarly, the EuroQol 5 Dimensions Visual Analog Scale score demonstrated a significant enhancement, rising from 73.17 ± 8.33 before treatment to 75.48 ± 7.22 after treatment (*t* = 2.434, *P* = .016). These results suggest that AK104 not only provides therapeutic efficacy but also contributes to improving the overall QoL in patients with advanced solid tumors who have failed PD-1/PD-L1 therapies.

### 3.7. Adverse events

**Figure 6.**
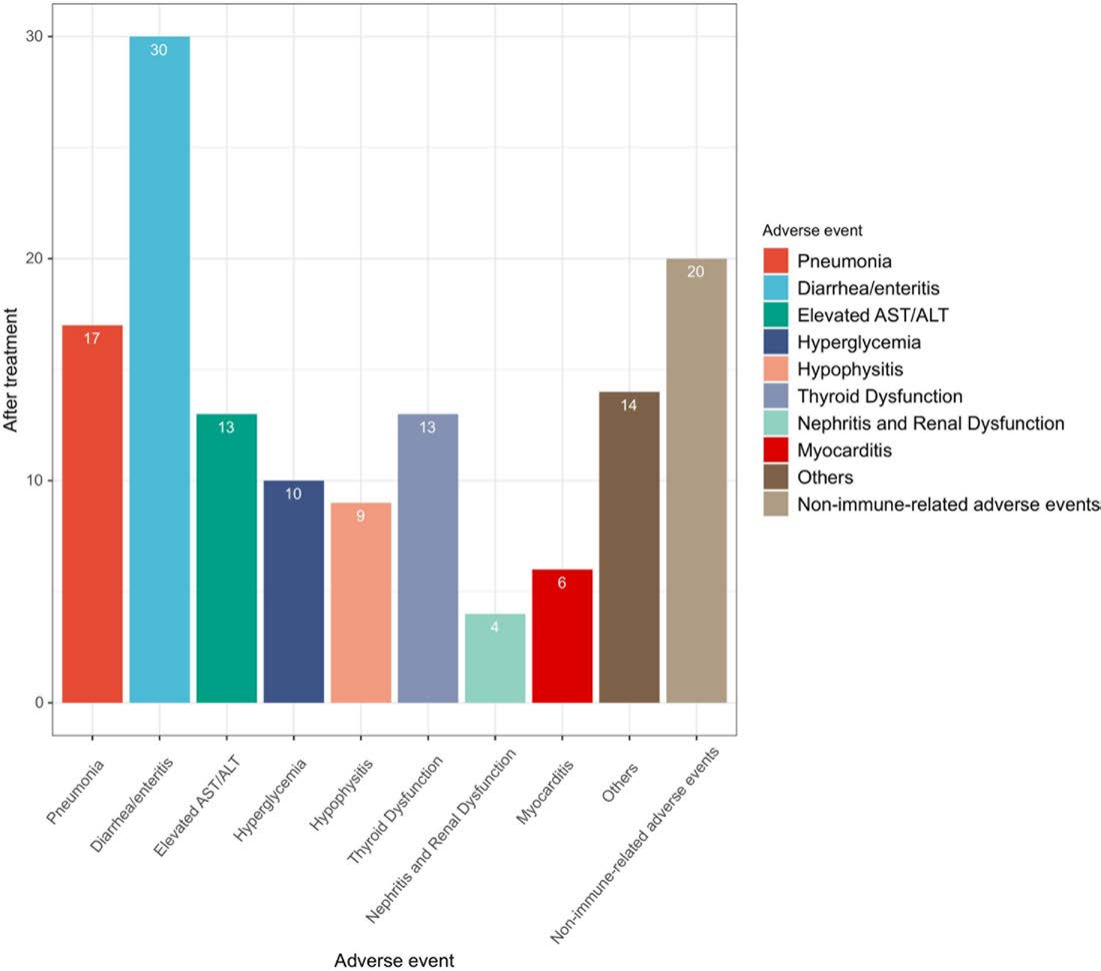
Adverse event after treatment with AK104. ALT = alanine transaminase, AST = aspartate transaminase.

The evaluation of adverse events following AK104 treatment revealed a predominance of immune-related adverse events, reported in 115 patients (85.19%) (Fig. 6). The most frequently observed events included diarrhea/enteritis (30 patients, 26.09%), pneumonia (17 patients, 14.78%), thyroid dysfunction (13 patients, 11.3%), and elevated aspartate transaminase/alanine transaminase levels (13 patients, 11.3%). Additional immune-related events, such as hyperglycemia, hypophysitis, nephritis/renal dysfunction, myocarditis, and other unspecified events, were also documented.

In contrast, nonimmune-related adverse events were reported in 20 patients (14.81%), further emphasizing the predominantly immune-mediated nature of the adverse events associated with AK104 treatment. These findings highlight the importance of vigilant monitoring and timely management of immune-related adverse events in patients undergoing AK104 therapy for advanced solid tumors. Moreover, they underscore the need for proactive strategies to mitigate treatment-associated toxicities and ensure patient safety.

## 4. Discussion

The findings from this retrospective cohort study provide valuable insights into the safety, tolerability, and efficacy of AK104, a novel PD-1/CTLA-4 antibody, in treating patients with advanced solid tumors who have previously failed PD/PD-L1 therapies. A comprehensive evaluation of AK104 in this patient population is critically important due to the unmet medical needs and limited treatment options for those experiencing resistance or intolerance to existing PD-1/PD-L1 inhibitors. This study enrolled 135 patients with various advanced solid tumors, and the results highlighted key baseline demographic characteristics and clinical outcomes following AK104 treatment. The findings suggest that AK104 has the potential to enhance clinical responses, prolong survival, and improve quality of life, although a range of adverse events were observed.

The high prevalence of advanced solid tumors, combined with the challenges of therapeutic resistance and adverse events associated with standard treatments, highlights the urgent need for alternative therapeutic strategies. The advent of immune checkpoint inhibitors targeting PD-1 and PD-L1 has revolutionized cancer therapy by harnessing immune system’s potent antitumor activity. However, a significant proportion of patients fail to derive durable benefits from PD/PD-L1 therapies, underscoring the need for novel immunotherapeutic options, such as dual PD-1/CTLA-4 blockade with AK104. The results of this study emphasize the pivotal role of AK104 in addressing the unmet needs of patients with advanced solid tumors refractory to standard PD/PD-L1 treatments.

The baseline demographic characteristics of the patient cohort reflect the heterogeneous nature of advanced solid tumors and the diversity of patients who faced therapeutic challenges following previous PD/PD-L1 therapy. The balanced distribution of sex, along with the average age, disease duration, BMI, ECOG score, and smoking and alcohol history, collectively describe a representative population with varied clinical backgrounds. The inclusion of patients with different tumor types, particularly non-small cell lung cancer, liver cancer, esophageal cancer, and stomach cancer, highlights the broad applicability of AK104 across diverse solid tumor types and its potential for widespread clinical impact.

The efficacy outcomes following AK104 treatment are particularly notable, demonstrating both partial response and stable disease in a significant proportion of patients. The observed partial response rate of 17.78% and disease control rate of 98.52% highlight the potential of AK104 to induce objective clinical responses and disease stabilization in patients with advanced solid tumors who have failed PD/PD-L1 therapies. These findings are consistent with preclinical and early clinical data highlighting the synergistic effects of dual PD-1/CTLA-4 blockade, leading to enhanced antitumor activity and favorable clinical responses.^[18]^ The ability of AK104 to achieve disease control and objective responses supports its potential as an effective therapeutic option for patients with therapeutic resistance or intolerance to existing PD-1/PD-L1 inhibitors.

While the efficacy outcomes are promising, the safety and tolerability profile of AK104 warrants careful consideration. The occurrence of immune-related adverse events, including fatigue, diarrhea, rash, and nausea, highlights the need for proactive monitoring and management of treatment-associated toxicities. The high incidence of immune-related adverse events, reported in 85.19% of patients, underscores the importance of vigilant surveillance and timely intervention to mitigate potential complications. Moreover, the prevalence of immune-related adverse events such as diarrhea, enteritis, pneumonia, and thyroid dysfunction emphasizes the need for comprehensive patient education, close monitoring, and tailored supportive care strategies to optimize the safety of AK104 therapy in this patient population.

The analysis of overall survival and progression-free survival (PFS) provides preliminary evidence of the potential clinical benefits of AK104 in patients with advanced solid tumors who have failed PD-1/PD-L1 therapies. The observed 1-year survival rate of 48.15% and 2-year survival rate of 31.11% suggest that AK104 may contribute to prolonged survival in this patient population. However, it is important to note that this study did not utilize Kaplan–Meier survival analysis, which is a more robust method for evaluating survival outcomes. The retrospective nature of the study and the absence of Kaplan–Meier analysis limit the strength of these conclusions. Future prospective studies with larger cohorts and more rigorous survival analysis methods are needed to validate these findings and gain a deeper understanding of the long-term survival benefits of AK104 in this setting.

The impact of AK104 on the QoL of patients with advanced solid tumors is a critical aspect of comprehensive cancer care. The observed improvement in QoL scores, as measured by validated assessment tools, highlights the potential holistic benefits of AK104 treatment beyond its direct antitumor effects. These enhancements in QoL suggest that AK104 may improve not only the physical, but also the psychosocial and functional aspects of patient experience, potentially leading to better overall well-being, functional status, and patient satisfaction. This comprehensive impact on QoL underscores AK104’s promise as a therapeutic intervention that targets both the tumor and the broader aspects of patients’ lives.

The balance between efficacy and safety is critical in the clinical evaluation of novel therapeutic interventions, particularly in patients with advanced solid tumors who have failed standard treatments. The findings from this study highlight the need for a nuanced approach to AK104 therapy, weighing its potential clinical benefits against the risk of immune-related adverse events. The observed efficacy outcomes, including the partial response and disease control rates, warrant further investigation in larger prospective studies to validate and expand upon the findings of this retrospective cohort analysis. Furthermore, a comprehensive assessment of immune-related adverse events and their management strategies is essential to optimize the risk-benefit profile of AK104 in clinical practice and to guide personalized patient care.

## 5. Conclusion

The safety, tolerability, and efficacy of AK104 highlight its potential as a valuable therapeutic option for the management of patients with advanced solid tumors who have failed PD-1/PD-L1 therapies. The demonstrated ability of AK104 to induce clinical responses, prolong survival, and improve quality of life underscores its promise as a viable treatment option. However, the necessity of proactive management of immune-related adverse events and the optimization of treatment strategies remains critical to maximizing its clinical utility. Continued exploration and validation in large, well-controlled clinical trials are essential to fully establish its therapeutic potential.

## Acknowledgments

The authors are grateful to all participants in the present study.

## Author contributions

**Conceptualization:** Jun Yi.

**Data curation:** Jun Yi, Huangpeng Yu, Yun Shu, Yingen Deng.

**Formal analysis:** Jun Yi, Huangpeng Yu, Yun Shu, Yingen Deng.

**Investigation:** Jun Yi, Huangpeng Yu, Yun Shu, Yingen Deng.

**Methodology:** Jun Yi.

**Writing – original draft:** Jun Yi.

**Writing – review & editing:** Huangpeng Yu, Yun Shu, Yingen Deng.
